# Rupture of pulmonary hydatid cyst in pediatrics: A cross-sectional study

**DOI:** 10.1016/j.amsu.2021.01.001

**Published:** 2021-01-08

**Authors:** Shadi Hamouri, Haitham Odat, Sebawe Syaj, Erich Hecker, Nasr Alrabadi

**Affiliations:** a Department of General Surgery and Urology, Faculty of Medicine, Jordan University of Science and Technology, Irbid, 22110, Jordan; b Department of Special Surgery, Faculty of Medicine, Jordan University of Science and Technology, Irbid, 22110, Jordan; c Faculty of Medicine, Jordan University of Science and Technology, Irbid, 22110, Jordan; d Thoracic Surgery Department, Thoracic Center Ruhrgebiet in Herne, University of Duisburg-Essen Teaching Hospital, Germany; e Department of Pharmacology, Faculty of Medicine, Jordan University of Science and Technology, Irbid, 22110, Jordan

**Keywords:** Hydatid cyst, Pulmonary, Children, Rupture, Cystectomy, Capitonnage

## Abstract

**Introduction:**

Pulmonary hydatid cyst is a parasitic disease causing an endemic and a health burden in many regions. Lung cysts are more common than liver cysts in children and patients may remain asymptomatic. Cyst rupturing is not uncommon, and it is considered the most feared complication. In this cohort study, we aimed to identify the risk factors related to cyst rupture in a Jordanian pediatric population.

**Methods:**

We retrospectively evaluated all pediatric patients who underwent cystostomy and capitonnage for pulmonary hydatid cyst between 2003 and 2020 at King Abdullah University Hospital.

**Results:**

We found 43 patients with a mean age of 13 ± 4 years who suffered from 61 pulmonary cysts. 55.6% of them were males. The most prevalent symptom was shortness of breath. The rupture rate for patients was 39.5%, and 29.5% for cysts. None of the patients with cyst rupture had an anaphylactic reaction. The left lower lobe was the most common location for both intact and ruptured cysts. 25.6% of the patients had giant cysts (>10 cm) with a mean of 7.4 cm for all cysts. Patients with intact cysts had higher-rates of cough (42.3% vs. 29.4%) and lower-rates of shortness of breath (34.6% vs. 52.9%) than patients with ruptured cysts, which were not statistically significant. Although statistically insignificant, patients with ruptured cysts tended to have multiple cysts in one lung (29.4% vs. 7.7%, p = 0.180), and more complication rates (29.4% vs 7.7%, p = 0.09). Both groups had almost identical IgG-ELISA positive results. We found no significant association between cyst rupture and age, gender, presenting symptoms, cyst size, cyst location, and rate of postoperative complications.

**Conclusion:**

The rupture of pulmonary hydatid cyst has clinical consequences in pediatric patients, further studies on larger populations are needed to identify factors that make patients more prone to rupture and prioritize them for clinical monitoring and management.

## Highlights

• Hydatid cyst rupture is a common serious complication in pediatrics with 39.5% of the diagnosed cases.
• About 25% of pediatric patients tend to develop giant cysts but the cyst size was not significantly associated with rupture.
• Pediatric patients with rupture pulmonary hydatid cyst have a low tendency to develop an anaphylactic reaction.
• Larger sample size is needed to define the risk factors and predictors of pulmonary hydatid cyst rupture.

## 1 Introduction

Hydatid cyst is a zoonotic disease caused by tapeworms from the Echinococcus genus. Four species of this genus are pathogenic, but most cases are caused by *E. granulosus* in its larval stage. Hydatidosis is endemic in many regions of the world, especially in rural terrains. Hydatid cysts develop more frequently in the liver, followed by the lung, and less commonly in the spleen, and kidneys. However, pulmonary cysts are the most common type of cysts in children [1,2].

If infected, patients may remain asymptomatic for months to years. Manifestations like cough, dyspnea, fever, nausea, vomiting, and thoracic deformations start to appear when the lung is infected. Most children and adolescents remain asymptomatic even with large lesions, due to the higher elasticity of their lung parenchyma [3,4]. The primary diagnosis is made through imaging. However, serological tests such as immunoglobulin G enzyme-linked immunosorbent assay (IgG ELISA) can be used to confirm the diagnosis [3,5].

In terms of treatments, surgery is the treatment of choice. However, pulmonary hydatid cysts are sometimes treated by oral benzimidazoles like albendazole [6]. On the other hand, spontaneous cyst rupture is the most feared complication; it leads to a sudden onset of chest pain, hemoptysis, cough, and fever. As well, rupture rates in a range between 24.1 and 61% have been reported in previous studies [7]. Hypersensitivity is also a serious possible outcome of rupture, and it manifests as urticaria, wheezing, or life-threatening anaphylaxis [3].

More studies are needed to investigate the characteristics of hydatid cysts and the risk factors for their rupturing in pediatric patients aiming to formulate a consensus and a prediction model to guide proper treatments. In this study, we aimed to identify factors that may influence or predict rupture of pulmonary hydatid cyst in pediatric patients. To our knowledge, it is the first study to be conducted among pediatric patients in Jordan.

## 2 Methods

This study was approved by the institutional review board (IRB) committee at King Abdullah University Hospital (KAUH). The study was registered with the Research Registry (researchregistry6358) in accordance with the declaration of Helsinki (https://www.researchregistry.com/browse-the-registry#home/). The study was conducted according to the guidelines of Strengthening the reporting of cohort studies in surgery (STROCSS) 2019 [8].The medical records of pediatric patients diagnosed with pulmonary hydatid cyst in the period from 2003 to 2020 were retrospectively reviewed. Demographics and clinical characteristics were extracted from KAUH's database which is a major tertiary and university hospital in northern Jordan. Information about the patients' age, sex, presenting symptoms, postoperative complications, recurrence, cyst features, hospital stay in days, and laboratory results were extracted and reported.

Complete blood count, enzyme-linked immunosorbent assay (ELISA), liver function test, chest X-ray, computer tomography (CT) of the chest with intravenous contrast, and abdominal ultrasound were also extracted and reported.

**Fig. 1:**
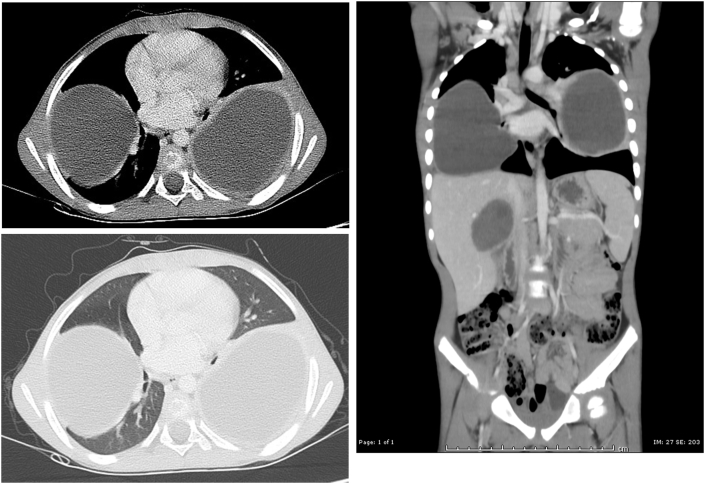
CT of the chest shows intact round, homogeneous, water-density with enhancing rim. **(A)** Axial mediastinal window, **(B)** Axial pulmonary window, **(C)** Coronal mediastinal window showing bilateral intact pulmonary and liver hydatid cysts.

**Fig. 2:**
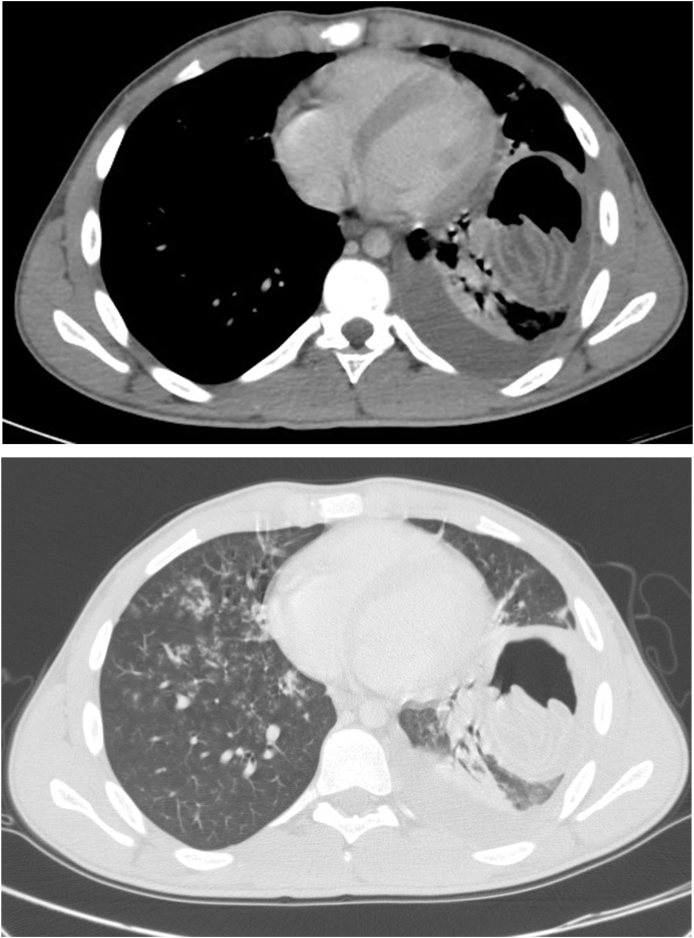
CT of the chest demonstrating a ruptured hydatid cyst at the left lower lung lobe. **(A)** The mediastinal window. It shows air-fluid level with crumpled endo-cyst membrane appearing as floating membrane associated with mild left-sided pleural effusion. **(B)** The pulmonary window. It shows ill-defined patchy ground-glass nodular opacities, and tree on bud appearance seen scattered on lung parenchyma suggestive of chemical pneumonitis.

Peripheral eosinophilia was defined as an eosinophil count in the peripheral blood of more than 0.5 × 10^9^/L. The diagnosis of pulmonary hydatid disease was mainly based on the CT findings by demonstrating a round, homogeneous, water-density with enhancing rim cyst in case of an intact cyst (Fig. 1 A, B, and C). The ruptured cysts usually posed a diagnostic challenge because they mimic lung abscess. The constellation of clinical features, laboratory as well as radiological studies was helpful in the diagnoses of a ruptured cyst (Fig. 2A and B).

**Fig. 3:**
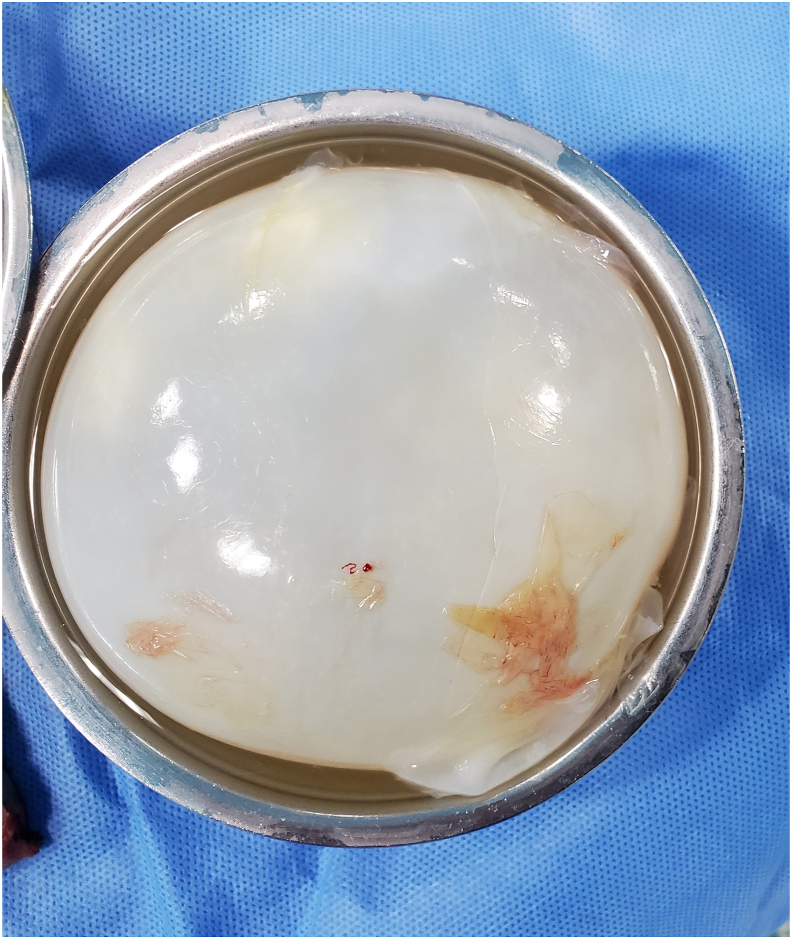
Macroscopic appearance of excised intact pulmonary hydatid cyst.

After 4–7 days of preoperative albendazole, all patients except one were treated with a parenchyma sparing resection cystostomy and capitonnage “Barret's procedure” (Fig. 3). The other patients underwent anatomical left lower lobe resection due to the destruction of the lobe. The surgical resection is performed through a posterolateral thoracotomy. The affected lung is usually isolated with an endobronchial blocker or by using double-lumen intubation. We used a 5% normal saline solution to protect the operative field. Postoperatively the albendazole is continued for 6 months in non-ruptured cysts and 1 year for ruptured or bilateral cysts to prevent a recurrence. The follow-up of patients was planned to be annual.

## 3 Statistical analysis

Frequencies/percentages for categorical variables and means/standard deviations for quantitative variables were reported in descriptive tables. Associations were investigated by comparing means in different groups using Student's t-test for quantitative variables. Pearson's χ^2^ test with Yates' continuity correction was used for categorical variables, and in cases of low cell count, a two-sided Fisher's exact test was used. Cysts larger than 10 cm in diameter were considered giant [9]. All statistical analyses were done using SPSS 26, and R statistical language version 4.0.3 [10].

## 4 Results

We retrospectively extracted data for 43 patients suffering from 61 pulmonary hydatid cysts. Their mean age was 13 ± 4 years (range 4–18 years) and 55.8% of the patients were males. Seventeen patients (39.5%) had at least 1 ruptured cyst, and only one patient had 2 ruptured cysts. Most patients had an intrabronchial rupture while 4 patients had a rupture into the pleural cavity. The commonest presenting symptom was shortness of breath (18 patients) and 3 patients experienced clinical recurrence. Moreover, 7 patients (16.3%) had cysts in both lung and liver, but no splenic or renal involvement was found. The mean hospital stay was 6.9 days. As well, none of the patients with a ruptured cyst experienced an anaphylactic shock. More details about the patients’ characteristics are displayed in Table 1.

**Table 1:** Patient demographics and clinical characteristics (n = 43).

Category Characteristics		Counts (%)
**Age**	13 ± 4	
**Sex**	Male Female	24 (55.8) 19 (44.2)
**Presenting symptoms**	Shortness of breath Cough Chest pain Fever Hemoptysis Asymptomatic	18 (41.9) 16 (37.2) 9 (20.9) 7 (16.3) 4 (9.3) 5 (11.6)
**Cyst side**	Unilateral Bilateral	35 (81.4) 8 (18.6)
**Lung side**	Left Right Bilateral	20 (46.5) 15 (34.9) 8 (18.6)
**# of cysts**	1 2 ≥3	29 (67.4) 10 (23.3) 4 (9.3)
**Liver involvement**	Yes No	7 (16.3) 36 (83.7)
**ELISA**	Positive Negative	28 (65.1) 15 (34.9)
**Eosinophilia**	Yes No	30 (69.8) 13 (30.2)
**Complications**	Air-leak Broncho-pleural fistula Pneumothorax Nil	3 (7.0) 1 (2.3) 3 (7.0) 36 (83.7)
**Single in one lung**	Yes No	34 (79.1) 9 (20.9)
**Multiple in one lung**	Yes No	7 (16.3) 36 (83.7)
**Single bilateral**	Yes No	5 (11.6) 38 (88.4)
**Multiple bilateral**	Yes No	0 (0) 43 (100)

Most patients had unilateral cysts (81.4%) where 20 patients had cysts in the left lung and 15 patients had them in their right lung, while 8 patients had cysts in both lungs. Twenty-nine patients had a solitary cyst and 14 patients had multiple cysts (2 cysts: 10 patients; 3 cysts: 4 patients). Of the 61 cysts, the left lower lobe of the lung was the most common location for cysts (42.9%) (Table 2). The mean maximum diameter of the cysts was about 7.4 cm where 25.6% of the patients had giant cysts (≥10 cm), 62.8% had large cysts (5–9 cm), and only 11.6% had small cysts (<5 cm).

**Table 2:** Cysts’ characteristics (n = 61).

Category Characteristics		Count (%)
**Cyst location**	LLL LUL RLL RML RUL	26 (42.6) 8 (13.1) 16 (26.2) 5 (8.2) 6 (9.8)
**Cyst status**	Intact Ruptured	43 (70.5) 18 (29.5)
**Maximal diameter (cm)**	7.4 ± 3.5	

LLL: Left lower lobe; LUL: Left upper lobe; RLL: Right lower lobe; RML: Right middle lobe; RUL: Right upper lobe.

As well, there were different scenarios to the cyst location and number; 34 (79.1%) patients had a single cyst in one of their lungs, other patients had multiple cysts in one lung (16.3%), some patients had a single cyst in each lung bilaterally (11.6%), and the last scenario is having multiple cysts in both lungs, which never happened in our cohort.

We divided the groups according to whether their cyst(s) were ruptured (39.5%) or intact (60.5%). The two groups had similar mean ages and gender distributions. Patients with intact cysts had higher rates of coughing (42.3% vs. 29.4%) and lower rates of shortness of breath (34.6% vs. 52.9%), but these differences were not statistically significant (Table 3). Although statistically insignificant, patients with ruptured cysts tended to have multiple cysts in one lung (29.4% vs. 7.7%, p = 0.180). Differences in lung side and number of cysts were insignificant as well.

**Table 3:** Patients characteristics according to cysts’ rupturing (n = 43).

Category Characteristics		Intact (%)	Ruptured (%)	P-value
**Total**		**26 (60.5)**	**17 (39.5)**	
Age		12.6 ± 3.4	13.3 ± 4.6	0.604
Sex	Male	14 (53.8)	10 (58.8)	0.428
	Female	12 (46.2)	7 (41.2)	
Cough	Yes	11 (42.3)	5 (29.4)	0.592
	No	15 (57.7)	12 (70.6)	
Fever	Yes	4 (15.4)	3 (17.6)	1.000
	No	22 (84.6)	14 (82.4)	
Shortness of breath	Yes	9 (34.6)	9 (52.9)	0.382
	No	17 (65.4)	8 (47.1)	
Chest Pain	Yes	7 (23.1)	3 (17.6)	0.722
	No	20 (76.9)	14 (82.4)	
Asymptomatic	Yes	4 (15.4)	1 (5.9)	0.623
	No	22 (84.6)	14 (94.1)	
Hemoptysis	Yes	2 (7.7)	2 (11.8)	1.000
	No	24 (92.3)	15 (88.2)	
Cyst side	Unilateral	21 (80.8)	14 (82.4)	1.000
	Bilateral	5 (19.2)	3 (17.6)	
Lung side	Left	12 (46.2)	8 (47.1)	1.000
	Right Both	9 (34.6) 5 (19.2)	6 (35.3) 3 (17.6)	
Single in one Lung	Yes	22 (84.6)	12 (70.6)	0.749
	No	4 (15.4)	5 (29.4)	
Multiple in one lung	Yes	2 (7.7)	5 (29.4)	0.180
	No	24 (92.3)	12 (70.6)	
Single Bilateral	Yes	4 (15.4)	1 (5.9)	0.633
	No	22 (84.6)	16 (94.1)	
# of cysts	1 2 ≥3	20 (76.9) 5 (19.2) 1 (3.8)	9 (52.9) 5 (29.4) 3 (17.6)	0.181
Liver involvement	Yes No	4 (15.4) 22 (84.6)	1 (6.7) 14 (93.3)	1.000
Eosinophilia (Y/N)	Yes No	19 (73.1) 7 (26.9)	11 (64.7) 6 (35.3)	0.807
ELISA	Positive Negative	17 (65.4) 9 (34.6)	11 (64.7) 6 (35.3)	0.964
Complications	Yes No	2 (7.7) 24 (92.3)	5 (29.4) 12 (70.6)	0.093

Additionally, we found that patients from both groups had almost identical ELISA test results (p = 0.964). Furthermore, 73.1% of patients with intact cysts had peripheral eosinophilia against 64.7% of patients with ruptured cysts (p = 0.807). As for postoperative complications, 3 patients had a pneumothorax, 3 patients had a prolonged air leak, and 1 patient had a broncho-pleural fistula. Patients with ruptured cysts had more complication rates (29.4% vs 7.7%) with marginal statistical significance (p = 0.09). Finally, we found no association between having cysts in the liver and eosinophilia or positive ELISA results.

The mean maximal diameter of the cyst was almost the same in each group (p = 0381). As well, the distribution of intact cysts was similar to that of ruptured cysts across different lobes; 32.6% of intact cysts occurred in the right lower lobe (RLL), while 16.7% of ruptured cysts occurred in RLL (Table 4).

**Table 4:** Cysts' characteristics according to cysts’ rupturing.

Cysts according to rupture		Intact cysts (%)	Ruptured cysts (%)	P-value
Total		43 (70.5)	18 (29.5)	
Cyst location	LLL LUL RLL RML RUL	18 (41.9) 6 (14.0) 14 (32.6) 3 (7.0) 2 (4.7)	8 (44.4) 2 (11.1) 3 (16.7) 2 (11.1) 3 (16.7)	0.444
Maximal diameter	(cm)	7.1 ± 3.2	7.9 ± 3.5	0.381

## 5 Discussion

Hydatidosis is a community health burden in many countries in the world, it's considered an emerging and re-emerging disease in some areas [6]. In this 17-year single-institution experience, we analyzed retrospectively the data for 43 patients of the pediatric population. We selected this age group because cysts grow rapidly in children and adolescents and can form giant cysts [4], which is reported to be related to the under-development of the immune system in these age groups [11]. Larger cysts are more commonly seen in the lungs than other organs, due to their elasticity and capacity for expansion. According to Usluer et al., giant cysts have a higher tendency for rupture, which is a dangerous complication and can lead to anaphylaxis or suffocation [4,12]. Giant cysts usually result from delayed diagnosis or treatment [13].

In our sample, the male-female ratio was 1.26, and there were no sex differences regarding the rupture of the cysts. This aligns with studies that found no association between sex and rupture in children [14] and adults [2]. On the other hand, Onal et al. [7] found an association between being male and having a ruptured cyst, but their sample had male predominance (70.9%).

Aldahmashi et al. [2] found a rupture rate of 37.8% in their sample of 148 cases of adult patients. As well, studies on pediatric patients found rupture rates of 38.1% and 34.2% [11,15]. In our study, we found that 39.5% of patients had at least 1 ruptured cyst which agreed with what is found in the literature regardless of the age groups.

Although the presentation of symptoms is usually delayed until adulthood due to the slow-growing nature of hydatid cysts [6], most patients (88.4%) from our sample presented with symptoms such as cough, chest pain, shortness of breath, fever, and hemoptysis. The remaining 5 patients who were asymptomatic got diagnosed incidentally. Shortness of breath was the most common presenting symptom, followed by coughing. In contrast with a recent study suggesting that pediatric patients with ruptured cysts had more symptoms than patients with intact cysts [16], the frequency of symptomatic patients was indifferent between the comparison groups in our study (p = 0.623). This could be related that the patients referred to the hospitals only when they have significant symptoms regardless of having intact or ruptured cysts.

Most of the cases had unilateral cysts in the left lung (46.5%), the remaining had unilateral cysts in the right lung (34.9%) or bilaterally (18.6%), and 67.4% of patients had a solitary cyst. These percentages were close to another radiological study of 102 pediatric patients [14]. At the level of cysts, 34 out of 61 cysts (55.7%) were in the left lung, and most of the cysts occurred in the left lower lobe (42.6%). This contrasts with studies that found more right lung involvement, most commonly in the right lower lobe, either in studies that excluded giant cysts [7], or those that included them [[13], [14], [16], [17]]. Onal et al. [7] investigated the relation between cyst location and rupture in pediatrics and they excluded patients with multiple cysts in one lung. They found that the right middle lobe and the lingula have significantly higher rupture rates than other locations, which suggests prioritizing patients who have cysts in these locations for surgery and management to avoid rupture. Such association was neither found in our study (p = 0.444), nor another study containing 145 cysts [14]. Interestingly, we found no difference in size between intact and ruptured cysts. Although larger cysts are reported to have a higher rupture rate, especially giant cysts [4].

Serological tests are useful in supporting initial radiological diagnoses, even though not all patients show a measurable immune response [1]. Peripheral eosinophilia is seen in 20–34% of patients [18]. Eosinophilia is not a diagnostic measure because of its limited specificity [19]. Surprisingly, 69.8% of our patients had eosinophilia, but without any relation with rupture (p = 0.559). This conflicting result needs more investigation by future studies.

IgG ELISA is the most reliable test with a sensitivity of 83.5% and a specificity of 99.5% [20]. Aydin et al. [21] addressed this test in the settings of rupture for adult patients (mean age = 27.7 ± 19.6 years). There was a strong association between having a ruptured cyst and testing positive for the IgG ELISA. They found that 90.6% of the ruptured cysts are tested positive. In our sample, we couldn't find such an association; the percentages of positive tests were almost identical between intact and ruptured cysts (65.4% and 64.7%, respectively, p = 0.964). The differences between our sample and the sample studied by Aydin et al. are the age (adults vs pediatrics) and the extrapulmonary involvement (they have 34.4% liver involvement in their patients vs. 16.3% in our sample). However, they found no association between IgG ELISA results and the age or extrapulmonary involvement. The association between ELISA and cyst rupture remains in need of further investigations.

According to Ramos et al. [19], IgG ELISA is more sensitive for patients with hepatopulmonary hydatidosis, this is because hepatic cysts are more likely to induce an immune response [22]. We have not found a difference in ELISA results between patients with liver involvement and others with solely pulmonary hydatidosis. This might be due to the small sample size, the liver involvement group contained only 5 patients, which is not enough to prove the difference statistically. Finally, the rupture was not correlated with a higher rate of postoperative complications (p = 0.093).

The strength of this study lies in focusing on the pediatric age group, which are more prone to develop giant cysts, which are more likely to rupture and cause dangerous consequences. The limitations include the retrospective nature of this study and the small sample size. Our sample size was 43, which got divided into 26 and 17 patients with intact and ruptured cysts, respectively. In our cohort the prevalence of pulmonary hydatid cyst rupture is relatively high (40%) and determining the predictors of rupture is critical in management plan. Shortness of breath, left lower lobe location, multiple cysts in one lung could possibly indicate higher probability for cyst rupture that may need immediate intervention. However, anaphylactic reaction is rarely encountered after cyst rupture. Based on the controversy between many treating physicians about the urgency of surgical intervention for pulmonary hydatid cyst, slow progression and benignity of the hydatid cyst and our findings in this study, we recommend conducting a multinational, multicenter prospective study to include a larger number of pediatric patients with pulmonary hydatid cysts to investigate all factors that can predict spontaneous cyst rupture and its complications. These include but not limited to size, intra-cystic pressure, laterality, lobar location, centrality, radiological features, peripheral eosinophilia, ELISA test and indirect hemagglutination test IHT. This will help the treating physician to stratify patients into two major groups. The first group are patients who need instant surgical intervention and the second group include patients who can wait for a period of time, in the latter the treating physician can try and investigate thoroughly the medical treatment as an alternative to surgery.

## 6 Conclusion

Pulmonary hydatid cyst rupture has clinical consequences in pediatric patients, further studies are needed to identify factors that make patients more prone to rupture and prioritize them for clinical management.

## Funding

Not Applicable.

## Ethical approvals

This work was approved by the institutional review board (IRB) committee at King Abdullah University Hospital (KAUH) under the approval number (8562020). The data can be available on request.

## Provenance and peer review

Not commissioned, externally peer-reviewed.

## Declaration of competing interest

The authors declared no conflict of interest.
